# In memoriam—Stephen I. Szára, D.Sc., M.D. (1923–2021)

**DOI:** 10.1038/s41386-021-01208-x

**Published:** 2021-10-23

**Authors:** Anna Wirz-Justice

**Affiliations:** Centre for Chronobiology, Psychiatric Clinic, University of Basel, Basel, Switzerland

**Keywords:** Neuroscience, Psychology

Stephen Szára, a Hungarian psychiatrist and chemist, often considered the “father of DMT” (N-N-Dimethyltryptamine), died on August 1, 2021. In the nineteen-fifties he became interested in the concept of model psychoses and the role of psychedelics in psychotherapy, and was the first to study the psychotropic effects of DMT, an alternative molecule he synthesized after Sandoz had declined to send LSD behind the iron curtain (arguing that such a powerful psychotropic drug might be dangerous if used unscrupulously). Szára recruited thirty volunteers, mostly young physician colleagues who received full psychedelic doses. He emphasized their common phenomenological experiences: “When these experiences, such as God, or strange creatures, appeared in our DMT studies, we did not philosophize about them but, as psychiatrists, we simply classified them as hallucinations.” He also theorized, “What DMT might do is to slow down and stop reality testing (via the fronto-parietal loop) and let the Default Mode Network release the stored images and symbols into the perceptual system. It is the brain that stores and releases archetypal images into our altered consciousness.” His later passion, arising out of these psychedelic drug studies, was conceptualising brain/mind interactions and developing computer simulations of multi-neuronal pathways: “by learning the mechanisms by which they affect the brain we may find keys to unlock the mysteries of the brain/mind relationship”.

Dr. Szára earned his DSc and MD from the Budapest University of Medicine, and established a research laboratory in a psychiatric hospital. However, three weeks after the Hungarian uprising in 1956, he left Hungary illegally, “with a briefcase containing a change of underwear, a couple of sandwiches, my two doctoral diplomas, test-tubes containing the newly discovered DMT, and 2 dictionaries: German/Hungarian and English/Hungarian, in search of a new life.” After some time in refugee camps, he contacted Professors Hoff, Arnold and Hoffman at the Viennese University clinic and traded some DMT for a chance to participate in their LSD study. In Berlin he was a guest researcher at the Free University with Professor Selbach, and in May 1957 attended the First International Congress on Psychotropic drugs in Milano. Later that year he took up a position at NIMH with Joel Elkes at St. Elizabeth’s Hospital in Washington, and subsequently explored the metabolism of DMT and related compounds in healthy and schizophrenic volunteers in Julius Axelrod’s lab.

This is when I met him, my uncle Pista, as a 17-year old AFS exchange student visiting Washington. He led me through his lab and talked about his experiments, and this single evening quietly inspired my own scientific research career. Who would have imagined that twenty years later I would also be fascinated by the neurotransmitter serotonin (genetic affinity?), become a visiting fellow at NIMH, share a house with him, and spend our daily commute to Bethesda talking about psychedelics?

In 1968, Dr. Szára joined the Center for Studies of Narcotics and Drug Abuse as Chief of Clinical Studies and conducted research on marijuana and THC. In that same year he was charged with reporting to the Director about the hippie scenes in SF, LA and NY, surely a sociological adventure for a psychopharmacologist. In 1974, when NIDA was created, he became Chief of the Biomedical Branch in the Division of Preclinical Research.

Dr. Szára was an Emeritus Fellow of the American College of Neuropsychopharmacology and Collegium Internationale Neuro-Psychopharmacologicum. He was also recipient of the Alcohol, Drug Abuse, and Mental Health Administration Administrator’s Meritorious Achievement Award and the Kovats Medal of Freedom from the American Hungarian Federation.

The complex neurotransmitter mechanisms underlying the subjective DMT experience are still not fully elucidated. As an endogenous compound in both plants and animals it has been considered a possible neuromodulator. Given its limited neurotoxicity, DMT may have therapeutic significance. It is indeed a tribute to his foresight, that nearly 70 years later, after a long hiatus where psychedelic drugs were deprecated and even outlawed, his prediction that these interesting molecules might be useful in psychotherapy is now undergoing a new wave of research.

